# Rift Valley Fever Vaccine Virus Clone 13 Is Able to Cross the Ovine Placental Barrier Associated with Foetal Infections, Malformations, and Stillbirths

**DOI:** 10.1371/journal.pntd.0004550

**Published:** 2016-03-31

**Authors:** Birgit Makoschey, Emma van Kilsdonk, Willem R. Hubers, Mieke P. Vrijenhoek, Marianne Smit, Paul J. Wichgers Schreur, Jeroen Kortekaas, Véronique Moulin

**Affiliations:** 1 Intervet International BV/Merck Sharp and Dohme (MSD) Animal Health, Boxmeer, The Netherlands; 2 Department of Virology, Central Veterinary Institute, Wageningen University and Research Centre, Lelystad, The Netherlands; The Kenya Medical Research Institute (KEMRI), KENYA

## Abstract

Rift Valley fever virus (RVFV) is a mosquito-borne pathogen that affects domesticated ruminants and occasionally humans. Classical RVF vaccines are based on formalin-inactivated virus or the live-attenuated Smithburn strain. The inactivated vaccine is highly safe but requires multiple administrations and yearly re-vaccinations. Although the Smithburn vaccine provides solid protection after a single vaccination, this vaccine is not safe for pregnant animals. An alternative live-attenuated vaccine, named Clone 13, carries a large natural deletion in the NSs gene which encodes the major virulence factor of the virus. The Clone 13 vaccine was previously shown to be safe for young lambs and calves. Moreover, a study in pregnant ewes suggested that the vaccine could also be applied safely during gestation. To anticipate on a possible future incursion of RVFV in Europe, we have evaluated the safety of Clone 13 for young lambs and pregnant ewes. In line with the guidelines from the World Organisation for Animal health (Office International des Epizooties, OIE) and regulations of the European Pharmacopeia (EP), these studies were performed with an overdose. Our studies with lambs showed that Clone 13 dissemination within vaccinated animals is very limited. Moreover, the Clone 13 vaccine virus was not shed nor spread to in-contact sentinels and did not revert to virulence upon animal-to-animal passage. Importantly, a large experiment with pregnant ewes demonstrated that the Clone 13 virus is able to spread to the fetus, resulting in malformations and stillbirths. Altogether, our results suggest that Clone 13 can be applied safely in lambs, but that caution should be taken when Clone 13 is used in pregnant animals, particularly during the first trimester of gestation.

## Author Summary

Rift Valley fever virus is a mosquito-borne virus that causes severe disease in young ruminants and occasionally humans. The virus is largely confined to the African continent, but mosquito vectors associated with transmission of RVFV are globally prevalent. There are no vaccines fully registered for use outside endemic areas. Clone 13 is a highly effective vaccine virus that was previously reported to be safe for cattle and sheep. We here report the results of safety studies performed with young lambs and pregnant ewes according to the guidelines from the OIE and regulations of the EP. In line with these guidelines and regulations, the studies were performed with an overdose. Our results confirm that Clone 13 can be safely applied in young lambs and show that the virus does not spread to the environment or contact lambs and does not revert to virulence upon animal-to-animal passage. However, inoculation of gestating ewes resulted in transmission of Clone 13 to the fetus and was associated with malformations and stillbirths.

## Introduction

Rift Valley fever (RVF) is a peracute or acute zoonotic disease of ruminants caused by the mosquito-borne RVF virus (RVFV), which belongs to the genus *Phlebovirus* within the family *Bunyaviridae*. Outbreaks of the disease are typically reported during climatic conditions that favour the breeding of mosquito vectors. During the first decades following its identification on a farm in the Great Rift Valley in Kenia in 1930, the virus was confined to the African continent. More recently, outbreaks have been reported on the Arabian Peninsula [1], Madagascar [2] and the Archipelago of Comoros and Mayotte in the Indian Ocean [3].

There is considerable variation in the susceptibility to RVFV between different animal species, with sheep, goats and cattle being the most susceptible. In these species, the disease is characterized by abortion, fetal malformation, neonatal mortality and liver damage.

Humans can become infected through contact with infected animal material or mosquito bite [4]. In humans, infections are usually unapparent or associated with flu-like symptoms that resolve without treatment. However, patients may develop severe complications such as haemorrhagic fever, meningoencephalitis and retinitis [5,6]. In a minority of patients (0.5–2%), the outcome of a RVFV infection is fatal [7].

Both inactivated and modified live-attenuated vaccines have been developed to control RVF epizootics. Inactivated vaccines require multiple doses in order to provide protection, whereas live vaccines generally require one dose to provide long-term immunity. In 1949, Smithburn and co-workers developed the first veterinary RVF vaccine by serial intracerebral passage of RVFV in mice [8]. The resulting Smithburn virus is a highly effective vaccine but can still cause liver damage [9] and is able to transmit to the fetus [10].

In the early 80’s Caplen and co-workers developed another live-attenuated vaccine, named MP-12 [11]. This vaccine virus was created by passage of the wildtype virus in the presence of the mutagen 5-fluorouracil, resulting in the accumulation of attenuating mutations on each of the three genome segments [12]. Although MP-12 vaccination results in low-level viremia, the vaccine was shown to be safe for cattle and sheep, even when applied during the second or third trimester of gestation [13–16]. In another study, however, MP-12 vaccination of ewes in the first and second trimester of gestation was associated with teratogenic effects [17]. In more recent studies in which 4 gestating ewes were vaccinated with either MP-12 or a recombinant derivative named arMP-12, no untoward effects were noted in the ewes, although 1 out of 4 ewes in each experiment was found to carry a dead fetus at the end of the experiment [13]. Since no MP-12 RNA was detected in these fetuses, the causes of death remained unknown. Altogether, these findings call for additional studies to further address the safety of MP-12 and recombinant derivatives.

Considering the remaining concerns about the safety of the Smithburn and MP-12 vaccines, the isolation of the naturally attenuated Clone 13 virus is considered one of the most important breakthroughs in RVF vaccinology [18]. The Clone 13 virus, isolated from a non-fatal human case in the Central African Republic, was shown to lack 69% of the gene encoding the nonstructural protein of the S genome segment (NSs). Extensive studies have demonstrated that the NSs protein counteracts host innate immune responses at different levels, *e*.*g*. by suppression of type-I interferon responses, and is thereby considered the major virulence factor [19–22]. The ability of the Clone 13 virus to induce protective immunity in lambs was recently confirmed in a vaccination-challenge study [23] and studies with calves and gestating ewes have supported the safety of Clone 13 [24–27]. In 2010, the Onderstepoort Biological Products (South Africa) company obtained a marketing authorization for Clone 13 and the vaccine was also applied in Botswana, Namibia, Zambia and Mozambique [28].

The recent introductions of two other arboviruses into Europe, Bluetongue virus serotype 8 [29–31] and Schmallenberg virus [32], have increased the concerns about potential incursions of RVFV. European-breed domesticated ruminants were shown to be highly susceptible to RVFV and several mosquito species that are associated with RVFV transmission in endemic areas are present in Europe. At this moment, several different vaccines, including Clone 13, are being considered as emergency vaccines for applications outside current endemic areas [28,33].

Here, we report the results of extensive safety studies performed with Clone 13 in lambs and pregnant ewes in line with guidelines from the World Organisation for Animal health (Office International des Epizooties, OIE) [34] and regulations of the European Directorate for the Quality of Medicines and Health Care (European Pharmacopeia, EP) [35]. In line with these guidelines and regulations, the studies were performed with an overdose. Two studies addressed characteristics in terms of dissemination, shedding, spreading (DSS study), and one study evaluated the potential of Clone 13 to revert to virulence (RTV study). Furthermore, we evaluate the ability of Clone 13 to cross the placental barrier by inoculating ewes at 50 or 120 days of gestation. Our results suggest that the Clone 13 vaccine is safe for young lambs, even after multiple administrations of an overdose via different inoculation routes, and does not spread to the environment or contact animals. Remarkably, inoculation during gestation resulted in vertical transmission of the virus to the fetus, which was associated with malformed lambs, stillbirths and precolostral antibodies. Untoward events were most prominent in ewes inoculated during the first trimester of gestation.

## Methods

### Ethics statement

All animal studies were approved by the Animal Ethics Committee of MSD Animal Health Boxmeer under permit number RVF 13.059 and were conducted in accordance with the Dutch Law on Animal Experiments.

### Virus

The Clone 13 virus [18] was kindly provided by Dr. M. Bouloy (Institut Pasteur, France) and was amplified in Vero cells, grown in 490 cm^2^ roller bottles.

### Overall study design

A total of 4 trials were performed with sheep. Trial 1 focussed on dissemination, shedding and spreading (DSS) of Clone 13, trial 2 on the risk of reversion to virulence (RTV), and trials 3 and 4 on safety of the virus for young lambs and pregnant ewes, respectively. Sheep were Texel/Swifter crossbreds and purchased in The Netherlands. Animals were free from RVFV and RVFV-specific antibodies at the start of the studies.

Work with live virus was performed by trained personnel in biocontainment facilities of MSD Animal Health (Boxmeer and Stevensbeek, The Netherlands) following appropriate biosafety procedures. The virus titres in the different vaccine preparations as well as the inoculation routes and vaccine volumes used in each study are presented in Table 1. In line with the regulations from the OIE and EP, all studies were performed with an overdose. This overdose was set at 10^7^ TCID_50_ as we anticipated that the maximum virus titre likely to be contained in 1 dose of the vaccine is 10^6^ TCID_50_.

**Table 1:** Inoculation routes and Clone 13 titers used in this study.

Study	Inoculation route^1^ and volume	Titer of inoculum [log_10_TCID_50_/ml]
DSS	SC 1.0 ml	7.9
	ID 0.2 ml	
RTV	SC 1.0 ml	7.4
	ID 0.1 ml	
Safety in lambs first vaccination	SC 2.0 ml	7.6
	ID 0.2 ml	
	IN 2x 0.5 ml	7.1
	IO 2x 0.5 ml	
Safety in lambs second vaccination	SC 1.0 ml	7.1
	ID 0.1 ml	
	IN 2x 0.5 ml	7.1
	IO 2x 0.5 ml	
Safety in pregnant ewes (~50 days after mating)	SC 1.0 ml	6.8
	ID 0.1 ml	
Safety in pregnant ewes (~120 days after mating)	SC 1.0 ml	7.0
	ID 0.1 ml	

^1^SC; subcutaneous, ID; intradermal, IN; intranasal, IO; intraocular

All treated and control animals were observed for local and systemic reactions and rectal body temperatures were recorded daily. Blood samples were collected at pre-set time points and tested for viremia and RVFV-specific antibodies.

### Dissemination, shedding and spreading (DSS) study

**Fig 1:**
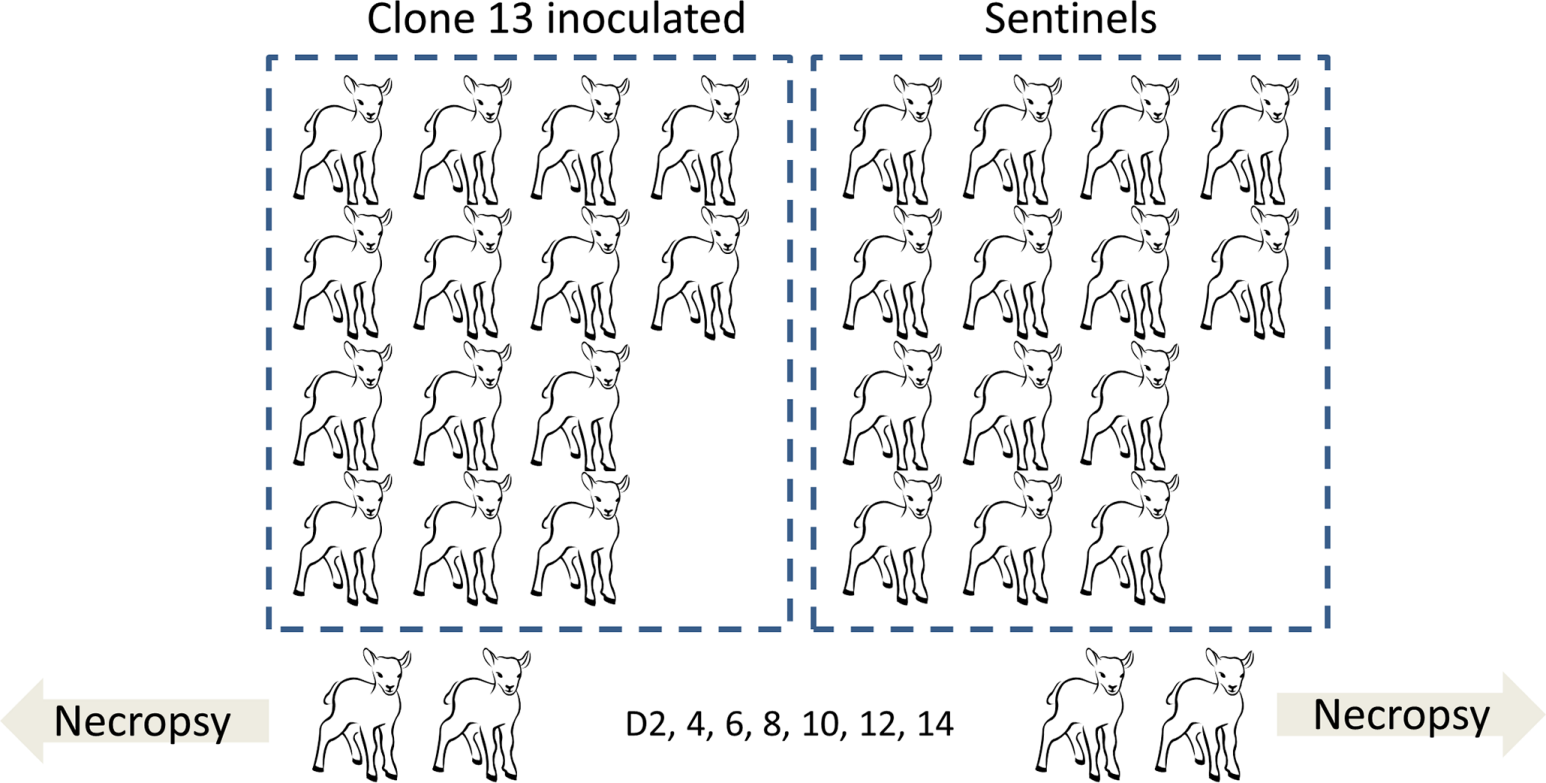
Schematic representation of the DSS study. Lambs (n = 14) were inoculated with Clone 13 via SC and ID route. A group of the same size functioned as sentinels. On days 2, 4, 6, 8, 10, 12 and 14, two lambs from each group were euthanized. The following samples were collected for detection of Clone 13: saliva, nasal mucus, lacrimal fluid, urine and faeces. During necropsy, gonades, kidney, adrenal gland, spleen, *Ln*. *ileocaecalis*, small and large intestines, bladder, pancreas, liver, lung, thymus, heart muscle, injection sites, *M*. *tripceps brachii*, Ln *prescapularis* left and right, salivary gland and brain samples were collected.

Thirty-two 8–10 week-old lambs were randomly divided into two groups of 14 animals. On day 0, lambs of group one were inoculated via the subcutaneous (SC) and intradermal (ID) routes with Clone 13. Animals from group 2 were left untreated and served as sentinel animals. Between days post inoculation (DPI) 2–14, two animals from each group were euthanized and necropsied every other day. The following samples were collected at necropsy: saliva, nasal mucus, lacrimal fluid, urine and faeces, gonades, kidney, adrenal gland, spleen, lymph node (Ln) *ileocaecalis*, small and large intestines, bladder, pancreas, liver, lung, thymus, heart muscle, injection sites, *M*. *tripceps brachii*, Ln *prescapularis* left and right, salivary gland and brain. The samples were evaluated for the presence of Clone 13 RNA using reverse transcriptase quantitative PCR (RT-qPCR) and virus isolation as described below. A schematic overview of the experiment is presented in Fig 1.

### Reversion to virulence (RTV) study

**Fig 2:**
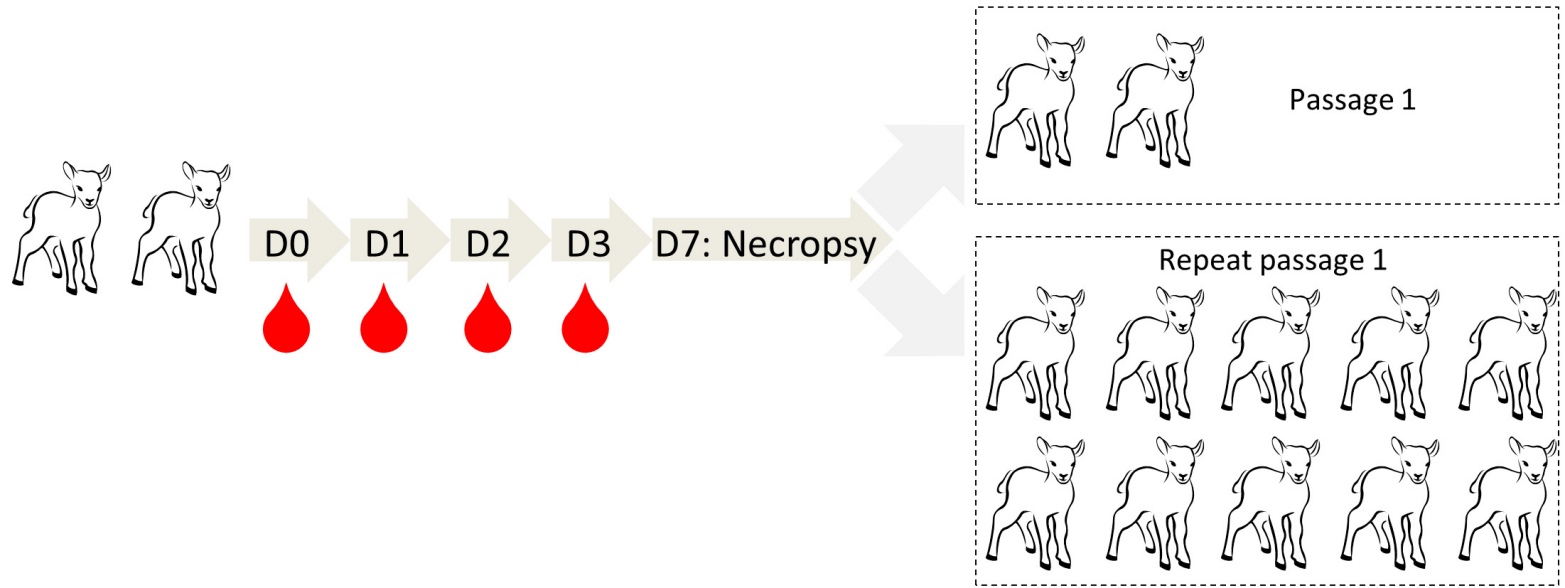
Schematic representation of the RTV study. Two lambs were inoculated via SC and ID routes and blood samples were collected on D0, 1, 2, and 3. On day 7, lambs were euthanized and prescapular lymph nodes were collected. Samples containing the highest amount of Clone 13 RNA were inoculated via the same routes into two naïve lambs (Passage 1). No virus RNA was detected in similar samples collected from these lambs. The passage was repeated with another group of 10 lambs (Repeat passage 1). In none of these lambs, Clone 13 RNA was detected.

A reversion to virulence study was performed with a total of fourteen 7–9 week old lambs. Two lambs were inoculated via SC and ID routes with Clone 13 (Table 1). Blood samples were subsequently collected at 0, 1, 2 and 3 days post inoculation and prescapular lymph nodes were collected at 7 days post inoculation upon necropsy. The plasma and prescapular lymph node homogenate samples with the highest virus content, as determined by RT-qPCR, were used to inoculate 2 naïve animals (first passage). Inoculation routes, sampling and testing regime of passage 1 was identical to passage 0. Finally, the passage was repeated with another group of 10 lambs. A schematic overview of the experiment is presented in Fig 2.

### Safety in lambs

Twenty 5-week-old lambs were randomly divided into two groups of 10 animals. Lambs of group 1 were inoculated with an overdose of Clone 13 by the SC, ID, intranasal (IN) and intraocular (IO) routes (Table 1). Inoculations were repeated two weeks later. Lambs of group 2 were left untreated. In addition to general clinical examination of the animals, the SC and ID injection sites were palpated to evaluate local reactions until two weeks after the second vaccination.

### Safety in pregnant ewes

**Fig 3:**
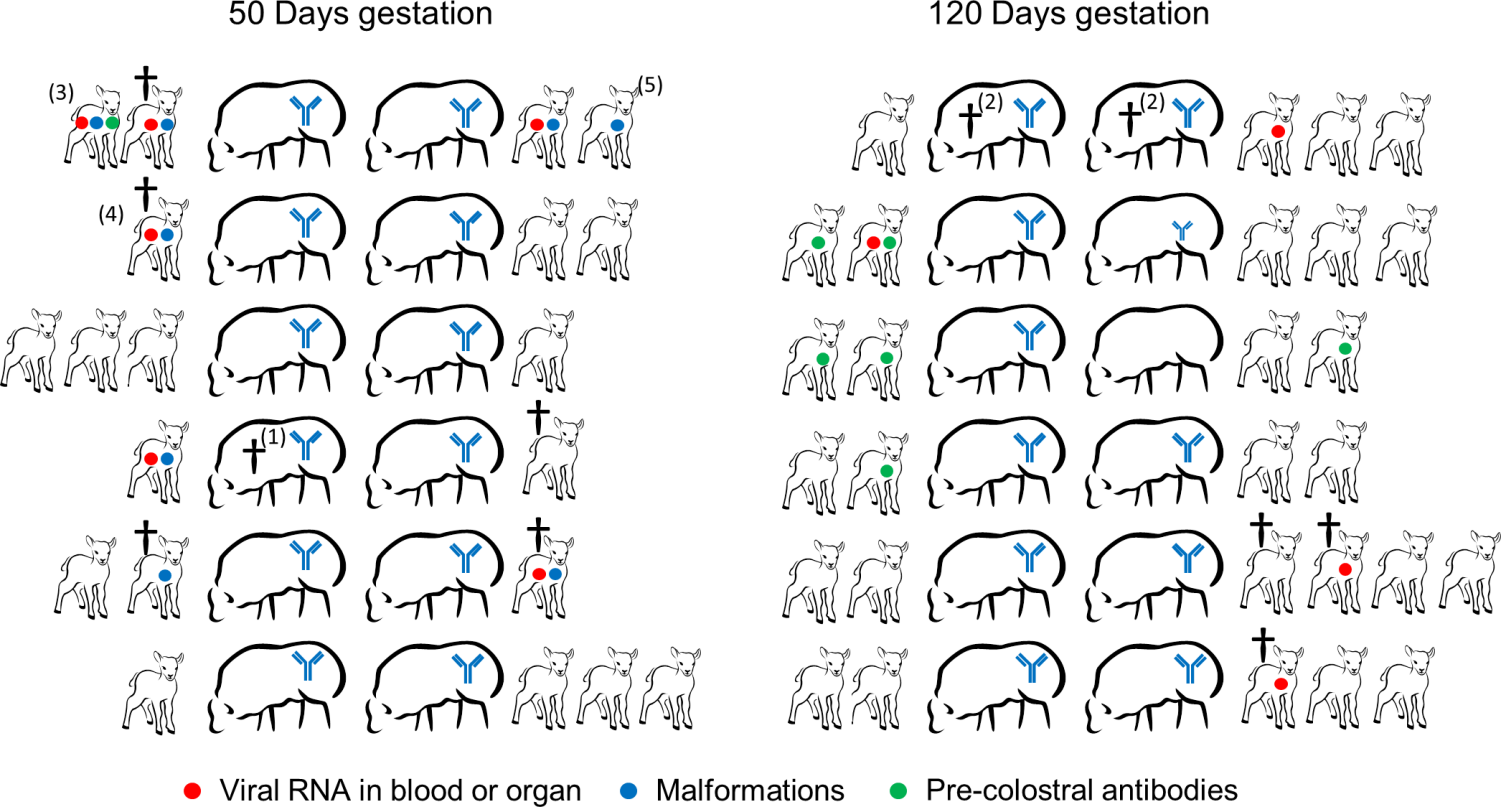
Schematic representation and major findings of the safety study with pregnant ewes. Ewes were inoculated with Clone 13 via SC and ID routes at 50 days or 120 days after mating. Ewes were monitored for clinical signs until delivery of the lambs, which were subsequently investigated for clinical signs, malformations, precolostral antibodies and presence of virus in blood and organs upon necropsy at the end of the study. Ewes seropositive by ELISA are indicated with a blue Ig symbol. The small symbol indicates a doubtful result. ^1^Ewe was euthanized after signs of imminent abortion; ^2^Ewe was euthanized because lambing had not occurred at the anticipated time point; ^3^Lamb was born alive, but showed signs of central nervous system dysfunction such as ataxic movements and disorientation and was euthanized to minimise suffering, ^4^Lamb corresponding to Fig 4A, 4B and 4D, ^5^Lamb corresponding to Fig 4E. Coloured dots refer to the following findings in lambs: viral RNA in blood or organ (red dots), malformations (blue dots) and precolostral antibodies (green dots).

Twelve approximately 50 days pregnant ewes (group 1) and another twelve approximately 120 days pregnant ewes (group 2) were inoculated with a high dose of Clone 13 via the SC and ID routes (Table 1). Two days post inoculation a blood sample was taken. Animals were subsequently monitored daily for general health and signs of abortion. Directly following natural delivery, precolostrum blood samples were taken from lambs borne alive and necropsy was performed on stillborn lambs. Lambs were euthanized at the end of the study when they were approximately two weeks old. All lambs, stillborn or alive, were examined macroscopically and histologically. Samples from brain, kidney, lung, liver, spleen and heart were tested for the presence of Clone 13 RNA by RT-qPCR and virus isolation. A schematic overview of the experiment is presented in Fig 3.

### Diagnostic assays

Viral RNA was isolated from EDTA blood samples and organ homogenates using the Roche MagNA Pure 96 (MP96) automated system for nucleic acid purification. The presence of Clone 13 RNA was assessed by Reverse-Transcriptase quantitative Polymerase Chain Reaction (RT-qPCR) using the Invitrogen SuperScript III Platinum One-Step qRT-PCR Kit (Invitrogen, 5791 Van Allen Way, Carlsbad CA, 92008, USA). Primers, probes and cycling conditions were applied as described previously [36]. C_q_-values and RNA copy numbers were calculated by the CFX Manager Software (Bio-Rad, 1000 Alfred Nobel Drive, Hercules CA, 94547, USA).

Samples that tested positive for Clone 13 RNA were used for virus titration on Vero cells. When virus was not detected, larger volumes of the inocula were incubated with Vero cells. The latter method increases the sensitivity of the virus isolation procedure, but does not allow the determination of virus titers. Isolation of Clone 13 was confirmed by immunofluorescence assays using a RVFV-specific monoclonal antibody (4D4, kindly provided by Dr. M. Bouloy, Institute Pasteur, France).

RVFV-specific antibodies in serum samples were detected by a commercial N-protein based ELISA kit (ID Screen Rift Valley Fever Competition Multi-species ELISA kit, IDvet, Montpellier, France) which was used according to the manufacturer’s instructions.

Tissue samples for immunohistochemistry (IHC) were fixed in formaline (4% buffered formaldehyde) and embedded in paraffin. Sections of 3.5 μm were mounted on silan coated slides and subsequently deparaffinised and dehydrated. Haematoxylin & Eosin (HE) staining was performed according to standard procedures. For IHC, sections were first incubated with peroxidase blocking solution (0.3% H_2_O_2_ in Methanol) for 5 min. A rabbit anti-myelin antibody (Myelin Basic Protein Ab-1, Thermo Scientific) was used as a primary antibody (1:200 in bovine serum albumin (1%) in TBS for 30 min) and a horseradish peroxidase labelled goat anti-rabbit antibody was used as the secondary antibody. Antibody binding was subsequently visualized using diaminobenzidine chromogen substrate (Envision System–HRP (DAP), Dako). Between incubations, sections were washed for 5 minutes in Tris-buffered saline (TBS) and cell nuclei were counterstained with Mayer’s haematoxylin for 10 seconds.

## Results

### Dissemination, shedding and spreading (DSS) of Clone 13 in lambs

To evaluate the extent of dissemination of Clone 13 within individual animals and to evaluate the potential ability of Clone 13 to spread to the environment and sentinel animals we performed a DSS study with lambs. Fourteen lambs were inoculated with Clone 13 and a group of equal size functioned as sentinels. Every other day, two inoculated lambs and two sentinel animals were euthanized and necropsied (Fig 1). No adverse reactions to the Clone 13 inoculation, such as reduced appetite, depression or elevated temperatures were observed and no Clone 13 RNA was detected in saliva, nasal mucus, lacrimal fluid, urine or faeces samples. Clone 13 RNA was detected in all but one of the plasma samples collected at dpi 2 (Table 2) and at the SC and ID injection sites. In addition, Clone 13 RNA was found in the draining lymph nodes of these animals. Notably, Clone 13 RNA was also found in two spleen samples and two liver samples albeit at very low amounts. Virus was isolated from 6 plasma samples collected at dpi 2 and 4 lymph node samples collected upon necropsy between dpi 2 and 6 (Table 2, indicated in bold). No infectious virus could be recovered from samples obtained >6 dpi. As expected, all sentinel animals remained free of Clone 13 RNA and did not develop RVFV-specific antibodies. Altogether, these results show that dissemination of RVFV Clone 13 was very limited and was largely restricted to the injection sites and the draining lymph nodes. Moreover, the virus was not secreted or excreted from inoculated animals and did not spread to sentinels.

**Table 2:** Detection of Clone 13 by RT-qPCR in samples taken from lambs during the dissemination, shedding and spreading (DSS) study^1^.

Animal no.	Plasma2 dpi	Necropsy on dpi			Injection site		Ln prescapularis		
			Spleen	Liver	left (SC)	right (ID)		left	Right
3164	-	2	-	-	6.2	-	-		6.8	
3184	4.8	2	-	4.4	8.0	4.0	6.8		**4.7**	
3178	4.3	4	-	-	-	6.5	**6.6**		7.0	
3179	4.6	4	-	-	6.6	-	7.5		**7.5**	
3163	4.4	6	-	-	-	6.0	**5.6**		7.1	
3168	4.5	6	-	-	-	-	7.0		6.4	
3161	4.7	8	4.7	3.5	4.5	3.6	6.2		6.5	
3182	**4.6**	8	-	-	-	-	7.8		7.7	
3169	4.5	10	4.4	-	3.9	-	3.9		5.4	
3165	**4.4**	10	-	-	5.4	4.2	-		-	
3172	**3.8**	12	-	-	-	-	-		5.7	
3176	**4.4**	12	-	-	4.7	5.3	5.3		5.2	
3174	**4.8**	14	-	-	-	-	4.5		-	
3189	**4.5**	14	-	-	-	-	-		-	

^1^RVFV specific RNA was measured by RT-qPCR in plasma samples taken two days after inoculation and from spleen, liver, both prescapular lymph nodes and the subcutaneous (SC) and the intradermal injection (ID) sites collected at necropsy at different days post inoculation (dpi). Results are depicted as log10 RNA copies/ml. A negative result in the RT-qPCR is indicated by the “-”sign. Samples from which virus was isolated are marked in bold. All samples collected from sentinel animals were RT-qPCR-negative and were therefore not included.

### Reversion to virulence (RTV) of Clone 13

To evaluate the potential of Clone 13 to gain virulence upon passage, a reversion to virulence study was performed. Two lambs were inoculated with Clone 13 via the SC and ID routes. As expected, Clone 13-specific RNA was detected in plasma samples collected one and two days post inoculation. On dpi 1, the first lamb contained 10^3.7^ RNA copies/ml plasma whereas the second lamb contained 10^3.9^ RNA copies/ml plasma. On dpi 2, viral RNA was only detected in plasma of the second lamb, with a titer of 10^3.3^ RNA copies/ml. In addition, Clone 13-specific RNA was detected in the left (lamb 1: 10^5.7^, lamb 2: 10^5.8^ RNA copies/ml) and right (lamb 1: 10^5.7^ and lamb 2: 10^5.5^ RNA copies/ml) prescapular lymph nodes collected during necropsy, which was performed 7 days post inoculation. To passage the virus to naïve animals, an inoculum was prepared of a plasma- and lymph node homogenate sample that contained the highest level of Clone 13 RNA. This sample was used to inoculate two lambs via SC and ID routes. In contrast to the plasma and lymph node samples obtained from the Clone 13 inoculated lambs, no Clone 13-specific RNA was detected in similar samples of passage 1 animals. The latter was repeated with another group of 10 lambs, with the same outcome (Fig 2). These results indicate that the risk of reversion to virulence of Clone 13 under natural conditions is very low.

### Safety of Clone 13 after administration of an overdose dose via different routes

The results of the DSS and RTV studies suggest that Clone 13 can be safely applied in lambs when applied via SC or ID inoculation routes. To further address the safety of Clone 13, we examined whether the vaccine is safe when applied at an overdose and after application of the virus via other routes (Table 1). As described in the M&M section, 10 lambs were inoculated with an overdose of Clone 13 via 4 different inoculation routes and 10 lambs were left untreated. Apart from elevated body temperatures in both groups on day 0 and 1, which was probably the result of mild stress due to handling, subsequent temperature measurements were within the physiological range. None of the lambs from either group displayed clinical signs during the course of the experiment. With a few exceptions, the injection sites were not visible and could not be palpated. Altogether these results indicate that Clone 13 is highly safe in lambs, even when a high dose is administered via four different inoculation routes.

### Safety of Clone 13 in pregnant ewes

The studies described above confirm that Clone 13 can be safely applied in lambs. However, the Clone 13 vaccine should preferably also be safe for pregnant animals. Safety of Clone 13 for pregnant animals was evaluated in a large trial with ewes at 50 or 120 days of gestation. As expected and similar to the lamb experiments, almost all blood samples (22 out of 24) were tested positive for RVFV RNA two days post Clone 13 inoculation. RNA levels were however too low to calculate copy numbers. With the exception of one, possibly two ewes that were inoculated at 120 days after mating, all ewes contained RVFV-specific antibodies in serum samples obtained two weeks post inoculation (Fig 3). None of the ewes developed fever or clinical signs throughout the course of the study.

One ewe in the group vaccinated at approximately 50 days after mating showed signs of imminent abortion at 76 days after inoculation. This ewe was euthanized for necropsy, which revealed that the foetus had the umbilical cord strangled around its legs, probably caused by the malformation of a foreleg, and did not contain brain tissue. In the remaining ewes, pregnancy continued until the anticipated date of birth. Interestingly, the number of stillborns and of malformed lambs was much higher than would be anticipated for a healthy herd (Fig 3). The proportion of stillborn lambs was 30% for the group inoculated at approximately 50 days after mating (including the foetus that was about to be aborted) and 11% for the group inoculated at approximately 120 days after mating. Necropsy was performed on two ewes that did not deliver at the anticipated time point. Based on the sizes and the general appearances and maturation of the foetuses it was concluded that the conception dates of these ewes were not correctly calculated. One lamb born from an ewe inoculated with Clone 13 at 50 days after mating showed typical signs of dysfunction of the central nervous system such as ataxic movements and disorientation. To prevent unnecessary suffering, the animal was euthanized. Another lamb in the same group had to be euthanized as well because of its overall poor condition. The average number of lambs per ewe was 1.7 for the group inoculated at 50 days after mating and 2.3 for the group inoculated at 120 days after mating.

**Fig 4:**
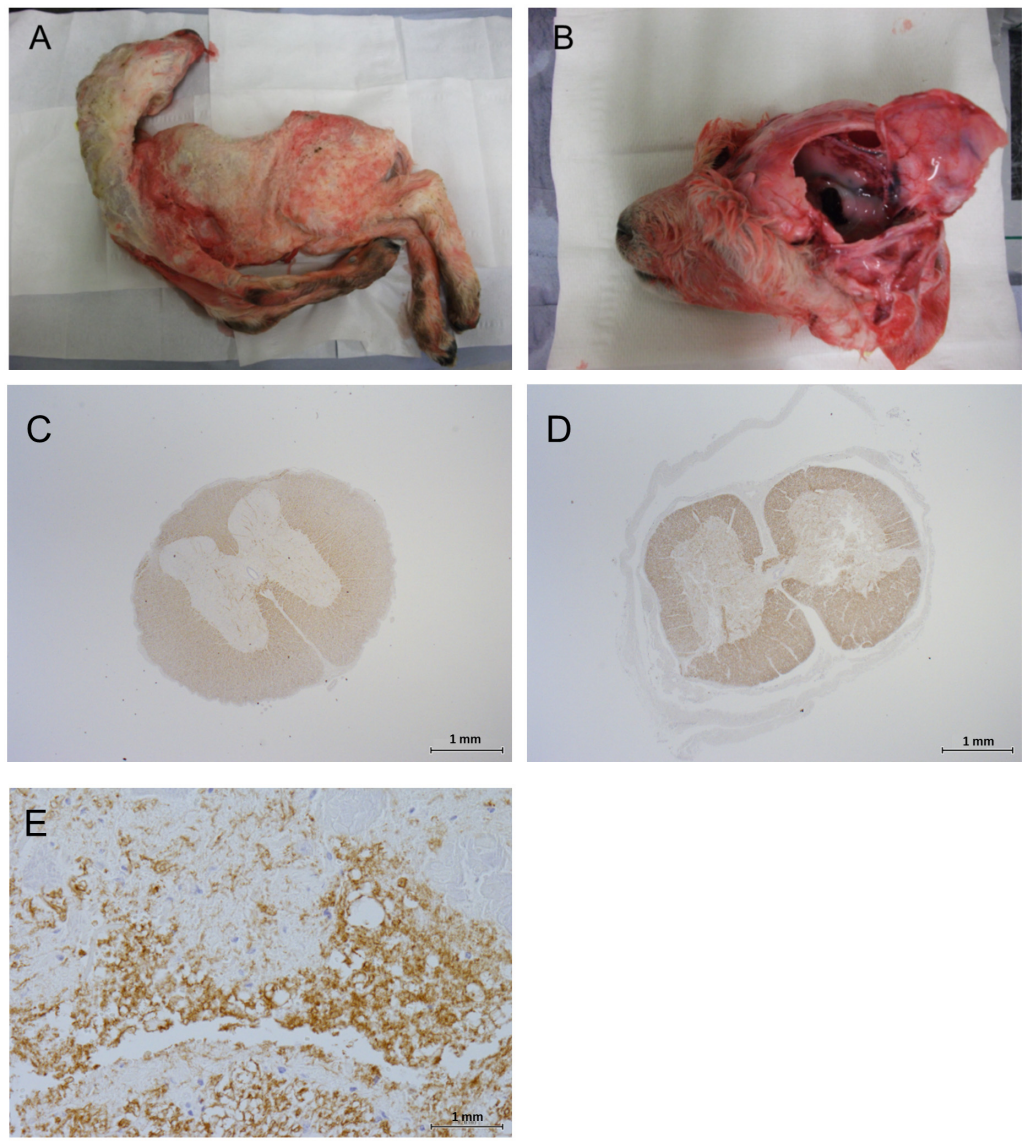
*In utero* infection of fetuses is associated with malformations of the skeletal- and central nervous system. (A) Arthrogryposis and scoliosis and (B) malformations of the central nervous system *i*.*e*. hydranencephaly and hypoplasia of cerebrum, and cerebellum in a stillborn lamb from an ewe vaccinated with the Clone 13 approximately 50 days after mating. (C) Myelin staining of the spinal cord from a fetus with normal morphology or (D and E) myelin staining of a spinal cord displaying hypoplasia of the grey matter (micromyelia) at (D) 20x magnification (E) 400x magnification. Myelin staining was performed with a rabbit anti-myelin antibody and a horseradish peroxidase-labelled anti-rabbit conjugate. The identities of the lambs are indicated by the footnotes in Fig 3.

No malformations were observed in lambs of ewes inoculated with Clone 13 at 120 days after mating. In contrast, as many as 7 lambs of 5 different ewes inoculated with Clone 13 at 50 days after mating showed malformations of the central nervous system such as hydranencephaly, hypoplasia of cerebrum, cerebellum and spinal cord and/or malformations of the musculoskeletal system such as; brachygnathia, arthrogryposis and scoliosis (Fig 4A and 4B). Histopathological examination of the spinal cord of these animals revealed hypoplasia of the grey matter, sometimes associated with vacuolation whilst in the cerebrum often accompanied with, edema, vacuolation (spongiosis) and liquefactive necrosis (Fig 4C and 4D).

Analysis of the lamb organs and plasma samples revealed that ten lambs were positive for Clone 13 RNA in the blood or one or more organ samples, six in the 50 days group and four in the 120 days group (Fig 3). Importantly, several precolostrum serum samples, one in the 50 days group and six in the 120 days group, tested positive for RVFV-specific antibodies (Fig 3).

## Discussion

Here, we report the results of studies on the safety of the Clone 13 vaccine virus for lambs, pregnant ewes and the ovine fetus. The ability of Clone 13 to disseminate, shed and spread to the environment was investigated as well as its ability to revert to virulence. Additionally, the safety of Clone 13 when applied at 50 or 120 days gestation was evaluated. The safety studies addressed different administration routes and repeated doses and were performed with an overdose as prescribed by the OIE and the EP. The combined results of all four studies demonstrate that Clone 13 is very well tolerated and safe for young lambs. However, inoculation of pregnant ewes was associated with foetal infections, malformations and stillbirths.

The observation that Clone 13 does not induce untoward effects in young lambs supports the notion that the vaccine can be applied safely in these animals. The dissemination, shedding and spreading (DSS) and reversion to virulence (RTV) studies also indicate that Clone 13 has a low risk of spreading from vaccinated animals. Viremia was very short-lived and of very low level, suggesting that the risk of Clone 13 transmission by mosquito vectors is negligible. According to OIE guidelines [34] and regulations of the European Directorate for the Quality of Medicines and Health Care [35], the potential of a live-attenuated vaccine virus to revert to virulence has to be studied even if the virus does not spread from animal to animal under natural conditions. Five animal-to-animal passages have to be performed in the most susceptible category of animals, following the route of vaccination that provides the virus with the most optimal conditions to revert to virulence. The Clone 13 virus was applied via the SC and ID routes. In the DSS study, the highest levels of viral RNA were found in plasma samples collected two days after inoculation and in prescapular lymph nodes taken between four and eight days after infection. Therefore, in the RTV study, blood samples were taken one, two and three days post infection and prescapular lymph nodes were collected seven days post infection. Although the Clone 13 virus could not be isolated from the collected samples, blood samples and lymph node homogenates were found to contain significant amounts of viral RNA. The samples with the highest levels of viral RNA were used to inoculate naïve animals. Analyses of similar samples subsequently collected from these animals did not reveal Clone 13 RNA, demonstrating that Clone 13 cannot be passaged from animal to animal. The inability to passage Clone-13 between young lambs strongly suggests that the risk of reversion to virulence of Clone 13 under natural conditions is negligible.

To assess the safety of Clone 13 for highly susceptible target animals, a study was conducted with very young lambs (five weeks old), which generally develop severe disease after infection with wildtype RVFV. Inoculation of these lambs with Clone 13 did not result in adverse systemic or local reactions. These results support the notion that the Clone 13 vaccine can be safely applied in young lambs. Although young lambs are highly susceptible to severe disease, the ovine fetus is the most susceptible. To investigate the ability of Clone 13 to cross the placental barrier, a large study was performed with ewes either at 50 or 120 days of gestation. As expected, none of the 24 pregnant ewes developed acute adverse reactions after administration of the Clone 13 vaccine. Remarkably, however, eight lambs born from ewes vaccinated at 50 days after mating showed malformations of the central nervous system, or the skeletal system, or both. These malformations are consistent with the consequences of intra-uterine virus infections and have been reported during RVF outbreaks in the field [37] and after vaccination with the live-attenuated RVF vaccine MP-12 [17]. Similar malformations may also occur after infection of pregnant ewes with Bluetongue virus, Border disease virus and Schmallenberg virus [38], however, these pathogens were all excluded as potential causes of the malformations observed in the present study, as no antibodies or viral RNA associated with these pathogens were detected. In addition, transplacental infection with Clone 13 was evident in 7 lambs by detection of RVFV-specific RNA in organ homogenates or blood samples and by detection of RVFV-specific antibodies in precolostral serum samples (Fig 3). Taken together, transplacental infection of Clone 13 took place in at least 6 out of the 12 ewes vaccinated approximately 50 days after mating and in 7 out of 12 ewes in the group vaccinated during the third trimester of pregnancy.

The results of the pregnant ewe study seem to contradict the results reported by Dungu and colleagues [25] who did not report any negative effect on the outcome of pregnancy after inoculation of Clone 13 in pregnant ewes, as well as the experiences from field studies with Clone 13 (26,27). The Clone 13 virus used in these studies originates from a master seed generously provided by Pasteur Institute (France). Therefore, a number of factors alternative to passage history, or combination thereof, may explain the apparent discrepancy between the study findings. These include the difference in numbers of animals used, the difference in breed of sheep and the virus dose.

The most plausible explanation for the differences in the study results is the virus dose, which was approximately 10 times higher in the study presented here. The previous studies with Clone 13 [25–27] as well as those with other live-attenuated RVF vaccines [39,40] were combined safety and efficacy studies, therefore animals were inoculated with a recommended dose, while the present study was designed as a dedicated safety study. In accordance with the guidelines of EU and OIE regulations, an overdose of Clone 13 was applied in the present work.

In addition to the functions of NSs in counteracting host innate immune responses, the NSs protein was also reported to mediate chromosome cohesion and segregation effects, suggested to be responsible, at least partially, for RVFV-mediated teratogenesis [41]. The finding that teratogenesis also occurs in the absence of NSs was therefore unanticipated. Further studies are warranted to elucidate the underlying mechanisms of Clone 13-mediated teratogenesis.

Based on the results obtained after overdose and repeated dose vaccination of young lambs, it can be concluded that Clone 13 has very favourable properties for use in lambs *i*.*e*. the virus does not cause acute local or systemic reactions, is not shed and does not spread to other animals and does not revert to virulence. However, caution should be taken when the vaccine is used in pregnant animals, especially during the first trimester of pregnancy, as Clone 13 may spread to the fetus and cause malformations and stillbirths. Our findings underline the value of safety experiments that comply with OIE guidelines and regulations described in the EP.
